# Phylogeny and In Silico Structure Analysis of Major Capsid Protein (L1) Human Papillomavirus 45 from Indonesian Isolates

**DOI:** 10.31557/APJCP.2020.21.9.2517

**Published:** 2020-09

**Authors:** Gita Widya Pradini, Edhyana Sahiratmadja, Sony Suhandono, Sunarjati Sudigdoadi, Muhammad Yusuf, Ade Rizqi Ridwan Firdaus, Herman Susanto

**Affiliations:** 1 *Department of Biomedical Science, Faculty of Medicine, Universitas Padjadjaran, Bandung, Indonesia. *; 2 *School of Life Science and Technology, Institut Teknologi Bandung, Bandung, Indonesia. *; 3 *Department of Chemistry, Faculty of Mathematics and Life Sciences, Universitas Padjadjaran, Bandung, Indonesia. *; 4 *Research Centre for Biotechnology and Bioinformatics, Universitas Padjadjaran, Bandung, Indonesia. *; 5 *Department of Obstetrics and Gynecology Dr. Hasan Sadikin General Hospital/Faculty of Medicine, Universitas Padjadjaran, Bandung, Indonesia.*

**Keywords:** Phylogeny, major capsid, L-1- HPV-45, Indonesia

## Abstract

**Background:**

Human papillomavirus (HPV)-45 genotype circulates in high percentage in Bandung area - Indonesia, after HPV-16 and HPV-18. The aim of this study was to analyse variations of major capsid (L1) HPV-45 and its phylogeny. Furthermore in silico protein structure and epitope prediction was explored.

**Methods:**

L1 gene of HPV-45 was amplified, sequenced and aligned. Phylogenetic tree had been built and compared with a complete L1 HPV-45 sequence. Structure and epitope prediction of L1 protein were then developed in silico.

**Results:**

Of 5 L1 HPV-45 sequences collected, we have detected one variant of sub lineage A2 which was considered as a new variant, and two variants of B2. Superimposition of structure of these two variants with reference showed very similar structure. Furthermore, seven amino acid substitutions were found within these L1 variants of which two substitutions might change the polarity of corresponding amino acid I329T and S383G. The S383G occurred in surface loop (HI-Loop) of new L1 HPV-45 variant.

**Conclusion:**

Similar structure of Indonesian variants indicates that amino acids variations do not affect the L1 structure. However, one substitution with altered amino acid polarity found within the area of surface loop suggests a potential impact in antibody recognition and neutralization.

## Introduction

Human papillomavirus (HPV) is a small, circular, double-stranded DNA virus with almost 8 kb genome size. Despite its small genome, the virus has diverse genetic classification with more than 170 HPV types have been identified (de Villiers, 2013). Of these, twelve HPV types which comprises of HPV-16, -18, -31, -33, -35, -39, -45, -51, -52, -56, -58, and -59 are classified as high-risk to cause cancer (Bouvard et al., 2009). Persistent infection with any of these high-risk HPV type is correlated with cervical cancer development (de Oliviera, et al., 2016). The classification of HPVs is based on open reading frame (ORF) of major capsid protein (L1), which is the most conserved gene within the genome. The difference within this gene with its closest isolate by more than 10% is defined as HPV genotype, between 2%-10% as HPV subtype and if the difference is less than 2% is defined as HPV variant (Bernard et al., 2006). Although HPV variants show highly similar sequence, the biological behaviours may distinct, for example, the difference in viral infectivity, persistence, antibody recognition and progression of cervical dysplasia to cervical cancer (de Oliviera et al., 2016; Bernard et al., 2005; Burk et al., 2013; Chen et al., 2014; Godi et al., 2016; Guan et al., 2015). 

The L1 protein is the major structural protein which encapsidate the HPV genome, and has a major role in infectious entry to the host. The protein form 72 pentameric capsomers and can self-assembled to construct an icosahedral capsid. The icosahedral structure has made L1 protein displayed evenly on the surface of the capsid, therefore, this L1 protein is highly immunogenic (Buck et al., 2013). The surface loop of the L1 protein may contain the sites of sequence variation among HPV types and also the location of dominant surface epitopes (Chen et al., 2000).

HPV-45 is one of the high-risk HPVs that contribute for about 5% of cervical cancer cases worldwide (Chen et al., 2014). Compare to other HPV types, HPV-45 together with the infection of HPV-16 and HPV-18 are suggested to have a relatively more carcinogenic potential than other high-risk HPV types (Guan et al., 2012). To date, it has been known that HPV-45 is classified into two major variants lineage A and B, and within this major lineage, the virus is grouped further into five variants of sub lineage A1, A2, A3, B1, and B2 (Guan et al., 2012). Some studies suggest that different HPV-45 variant may have been linked to different risk for cervical cancer development as well as protection from currently available HPV vaccine (Chen et al., 2014; Godi et al., 2016; Xi et al., 2014). Unlike HPV-16 and HPV-18, the geographic distribution of HPV-45 variant lineage varies around the world. Information about HPV-45 variant distribution may have beneficial impact for cervical cancer prevention strategies. HPV-45 is the third most prevalent HPV found in cervical cancer after HPV-16 and HPV-18 in Bandung, West Java province, Indonesia (Tobing et al., 2014). To our knowledge, studies describing on genetic variation and lineage of HPV-45 from Indonesia are limited. The purpose of this study was to analyse nucleotide variations of complete HPV-45 major capsid protein (L1) from Indonesia and its phylogeny. Furthermore, in silico protein structure and epitope prediction was explored.

## Materials and Methods

*Sample Collection*

DNA analysed in this study were obtained from cervical cancer tissue, and further underwent HPV Genotyping Test (Linear Array Genotyping Test, Roche Molecular System, Inc., Branchburg, NJ, USA). Only samples with confirmed HPV-45 were included in this study. The study protocol was approved by Ethical Committee of Faculty of Medicine, Universitas Padjadjaran no. 289/UN6.C2.1.2/KEPK/PN/2013. 

*PCR amplification and sequencing*

Amplification of L1 HPV-45 ORF (±1600 bp) was performed using sense primer 5’-GTGGCCTAGTACATCTCCTA-3’ and antisense primer 5’-TCATACATACACGCACGC-3’. 10mMol of each sense and antisense primer were added into the PCR mixture containing of 12.5 µl PCR mix (KAPA 2GFAST Ready Mix, KAPABIOSYSTEM, USA), 20ng template DNA, and PCR grade water added for a total reaction volume of 25 µl. The cycling condition was as followed: initial denaturation at 94°C for 3 minutes; amplification phase for 30 cycles of 94°C for 30 seconds, 58°C for 30 seconds, 72°C for 30 seconds; followed by 1 cycle of 72°C for 10 minutes (PalmCycler thermal cycler, Corbett Research). PCR products were confirmed by 1% agarose electrophoresis and visualized under UV light (GelDoc, BioRad). 

All confirmed PCR products were sent for purification and sequencing, of which samples were sequenced in both direction sense and antisense (1st Base, Malaysia). 

*Sequence analysis and phylogenetic tree construction*

The L1 HPV-45 sequences were edited to exclude PCR primer binding sites and corrected manually (BioEdit 7.2), and the obtained sequences were further aligned (MEGA 7.0.) (Tamura et al., 2013). Nucleotide variations of L1 HPV-45 from this this study were then compared to reference sequence published in GenBank (accession number X74479.1). Any nucleotide change in L1 HPV-45 sequence was explored for its implication. 

To construct phylogenetic tree, additional open reading frame (ORF) of L1 HPV-45 sequence was downloaded (http://www.ncbi.nlm.nih.gov/), and the referenced source list sequences used in this study was provided in Suppl. 1. The phylogenetic tree was further constructed (MEGA version 7.0) (Tamura et al., 2015), using maximum likelihood method and fit-substitution model. Tamura three-parameter and the non-uniformity of evolutionary rates among sites were modelled by a discrete Gamma distribution (T92+G). A 1,000 bootstrap replicates were performed to assess the confidence of branching pattern of the tree. 

*Homology Modelling*

The template structure for homology modelling was determined by using BLAST-x (blast.ncbi.nlm.nih.gov). The best template structures were then downloaded from Protein Data Bank (RCSB.org). The nucleotide of target sequences were translated to amino acid sequences by using ExPASy Translate Tool (web.expasy.org/translate/). The target and template sequences were aligned using Biovia Discovery Studio, by using structure oriented alignment (Dassault Systémes BIOVIA, 2017). The alignment sequences were used as the input file of the Modeller 9.19 software (Sali et al., 1993). Twenty homology models were generated from each target sequences, and ranked by their DOPE score. The best models were further refined and energy minimized by steepest descent method using Modeller 9.19. Furthermore, the refined models were assessed by Rampage Ramachandran plot analysis (Lovell et al., 2002) and DOPE graph profile (Sali, et al., 1993). 

*Epitope Predictions*

Structure-based epitope prediction had been made using Ellipro (http://tools.immuneepitope.org/tools/ElliPro) (Ponomarenko, et al., 2008). The predicted epitopes were mapped on the pentamer protein structure and visualized by using Biovia Discovery Studio (Dassault Systémes BIOVIA, 2017).

## Results

*L1 HPV-45 Sequence Variations*

In total, 5 cervical cancer tissues infected by HPV-45 in single or multiple infection were included. The histopathological diagnosis and the distribution of HPV infection among isolates was shown in Table 1. Of 5 L1 HPV-45 sequences analysed (1,611 bp length), 3 HPV variants were identified. Intratype nucleotide variations were calculated as previous study with the presence of insertion/deletion was counted as single occurrence regardless the number of nucleotides changed (Chen et al., 2009). There were 21 sites of nucleotide variations discovered (1.3%), of which seven variants resulted in amino acid substitutions (non-synonymous mutation) (Table 2). One sample (BDG-163) was consider as new variant with single nucleotide changed in nucleotide position 6705 from G to C compared to its closest isolate which originated from Costa Rica, Central America (Genbank accession number: EF202159.1). 

*Phylogenetic analysis*

**Figure 1:**
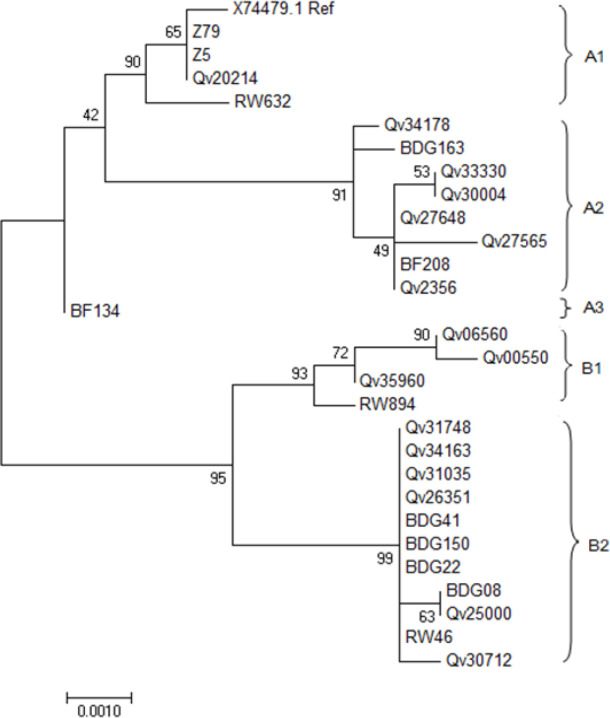
Phylogenetic Tree Comparing HPV-45 L1 ORF from Indonesian Isolate (BDG08; BDG22; BDG41; BDG150; BDG163). Sublineage (A1, A2, A3, B1, and B2) attribution is based upon whole-genome sequencing (Chen Z et al., 2013). The evolutionary history was inferred by using the Maximum Likelihood method based on the Tamura 3-parameter model (Tamura et al., 2013). Numbers near branches indicate bootstrap percentages (1,000 replications). Scale bar shows nucleotide substitutions per site. Evolutionary analyses were conducted in MEGA7 (Tamura et al., 2013)

The HPV 45 L1 from Indonesian isolates were distributed into two groups in phylogenetic tree (Figure 1) that corresponded to the previously described sub lineage A2 and B2 (Chen et al., 2013). The new HPV-45 variant (BDG-163) was clustered into sub lineage A2, while 4 others (BDG-150, BDG-08, BDG-22, BDG-41) were clustered into B2 sub lineage. Sample BDG08, BDG22, BDG41 were identical and 100% similar with some samples from Costa Rica and Rwanda (Figure 1). Sample BDG-08 was identical with another sample from Costa Rica (Qv2500). BDG-163 was considered as a new variant with a difference of 0.1 % in L1 ORF, with the closest isolate from Costa Rica. 

*Homology Modelling*

Three crystal structures were found at the top ranking templates in terms of percentage identity and query cover. Crystal structure of L1 papillomavirus type 18 (PDB ID 2R5I) was selected as the best template structure with 82% identity and 84% query cover. Crystal structure of L1 papillomavirus type 59 (PDB ID 5J6R) showed 80% identity and 88% query cover, and crystal structure of L1 papillomavirus type 16 (PDB ID 1DZL) showed only 67% identity and 89% query cover. 

**Figure 2:**
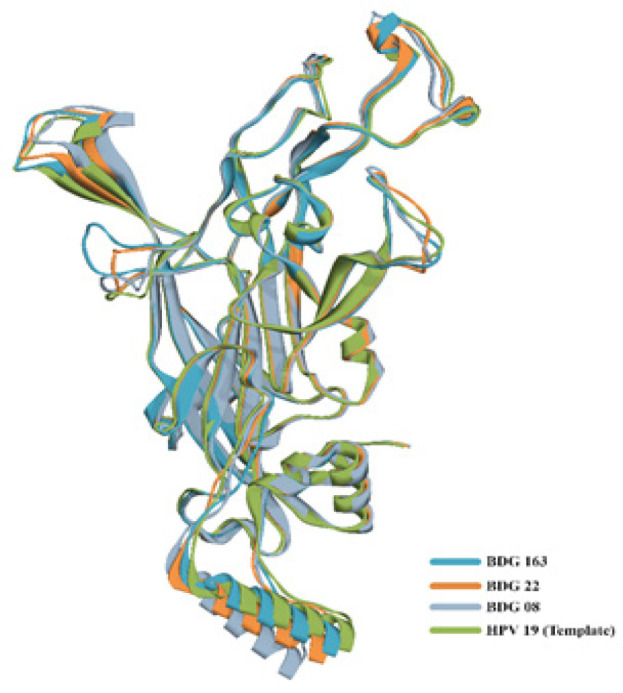
Predicted Structure of Major Capsid Protein HPV-45 Shows are no Major Different in the Structure of Bandung Indonesia Variants

Twenty homology models from each sequence had been constructed. Protein model with the best DOPE score was further refined and optimized. Stereochemical quality of the models in term of Phi-Psi plots (Ramachandran Plot) showed that 99.1% residues fall in the favorable and allowed region and the rest of 0.9 % residues in the disallowed region. The structure of all three variation sequences of HPV-45 L1 were modelled. In general, these structures were geometrically similar, as shown in Figure 2.

*Structural Epitope Prediction and Amino Acid Mutation*

**Figure 3:**
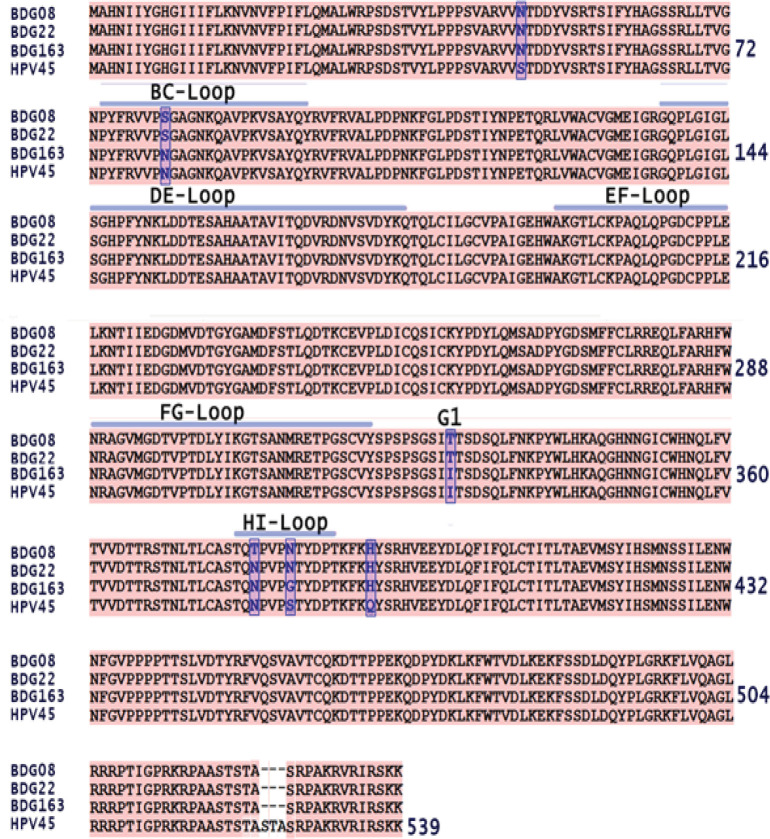
Sequence Alignment of Bandung L1 with Reference HPV-45 L1 (GenBank accession number X74479.1) along with external loops of HPV 45 L1 which mapped according to Bishop et al., 2007. Non-synonymous amino acid mutation were highlighted blue, external loops of HPV L1 were marked with blue line. The I329T mutation occurred in G1-β sheets

**Figure 4:**
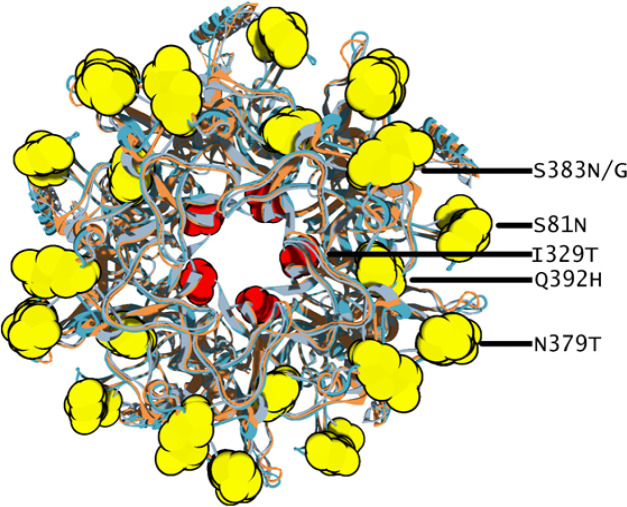
The Pentamer Structure of All Bandung HPV 45 L1 (Top Surface Region). Altered amino acid residues were showed in yellow and red color within the pentamer. The yellow colors (S81N, N379T, S383N, and Q392H) indicate similar amino acid polarity, and the red color (I329T) indicate altered amino acid polarity. The S383G mutation result in altered polarity and it occur in isolate BDG-163, in this figure it is mapped together with S383N since it takes place within the same number of amino acid sequence

Epitope prediction produced ten putative linear B-cell epitopes, which comprise of 9 - 36 amino acid residues (Suppl. 2). There were four variable epitopes, in which two of them were located on the surface area (Table 3). Seven amino acid mutations along with external loops of HPV 45 L1 were highlighted accordingly (Bishop et al., 2007) (Figure 3) and mapped to pentamer structure of HPV 45 L1 (Figure 4). Mutations of S49N, N81S, N379T, S383N and Q392H might not affect the antibody binding, since the structural properties between asparagine (N), threonine (T), serine (S), and glutamine (Q) are similarly polar. The change of polarity in amino acid side chains are reflected in I329T and S383G mutations. A nonpolar isoleucine (I) was changed to a more polar threonine. However, this mutations is not occurred within the exposed surface area (Figure 4). It is worth noting that the mutation of S383G, which changed the polarity of side chain, is also located on the surface epitopes and presents in the isolates of BDG-163, a new L1 HPV-45 variant (Table 3 and Figure 4). 

**Table 1:** Histopathological Diagnosis and the Distribution of HPV Infection among Isolates from Bandung

Sample Code	Histopathology	HPV Infection
BDG-8	Squamous cell carcinoma	HPV-16,-45, -52 multiple infection
BDG-22	Adenocarcinoma	HPV-16,-18,-45,-52 multiple infection
BDG-41	Squamous cell carcinoma	HPV-16,-18,-45,-52 multiple infection
BDG-150	Squamous cell carcinoma	HPV-16,-45 multiple infection
BDG-163	Squamous cell carcinoma	HPV-45 single infection

**Table 2:** HPV-45 Variants of Indonesian Isolates

Nucleotide	56	57	57	58	61	62	62	63	64	65	66	66	66	66	67	68	68	68	69	70	70	70	70	70	70	70	70	70	71
	75	12	71	41	53	13	70	6	29	15	65	76	77	87	5	16	37	61	51	90	91	92	93	94	95	96	97	98	43
X74479.1 (Ref)	G	T	A	A	A	G	T	A	G	T	A	A	G	C	G	A	C	G	A	T	C	T	A	C	T	G	C	A	G
	#		#							#	#	#	#		#														
Reference codon	A		A							A	A	A	A		G					TCT			ACT			GCA			
	G		A							U	A	G	G		U														
	C		U							U	U	U	U		A														
Altered codon	A		A							A	A	G	A		C														
	A		G							C	C	G	A		U														
	C		U							U	U	U	U		A														
Non synonimous mutation	S		N							I	N	S	S		Q														
	49		81							329	379	383	383		392														
	N		S							T	T	G	N		H														
A2																													
BDG-163	A	.	.	.	G	A	G	T	.	.	.	G	.	.	C	G	.	A	G		(-)			(-)			(-)		.
B2																													
BDG-08	A	C	G	G	.	.	.	.	A	C	C	.	A	T	C	G	A	A	.		(-)			(-)			(-)		A
BDG-22/41/150^	A	C	G	G	.	.	.	.	A	C	.	.	A	T	C	G	A	A	.		(-)			(-)			(-)		A

Note. The nucleotide and amino acids position of detected changes were written at the top row. The nucleotide numbering refers to X74479.1. ^Sequences from isolate BDG-22, BDG-41 and BDG-150 are identical. (#) indicates nucleotide change resulting in amino acid substitution (non-synonymous mutation); (.) indicates nucleotide match with reference; (-) indicates nucleotide deletion, there were deletion of 9 bp (TCTACTGCA) from nt7090 to nt7098.

**Table 3:** Putative Linear B-cell Epitopes of HPV-45 L1 from Indonesian Isolates and Reference

Epitopes	Isolate	Amino Acid Chain Sequence	Amino Acid Sequence
Epitope 1 (non-surface exposed)	Ref	46-62	R V V S T D D Y V S R T S I F Y H
	BDG08	46-60	R V V N T D D Y V S R T S I F - -
	BDG22	46-61	R V V N T D D Y V S R T S I F Y -
	BDG163	46-61	R V V N T D D Y V S R T S I F Y -
Epitope 2 (non-surface exposed)	Ref	326-334	G S I I T S D S Q
	BDG08	326-334	G S I T T S D S Q
	BDG22	326-334	G S I T T S D S Q
	BDG163	326-334	G S I I T S D S Q
Epitope 3 (surface exposed)	Ref	77-90	R V V P N G A G N K Q A V P
	BDG08	77-89	R V V P S G A G N K Q A V -
	BDG22	77-90	R V V P S G A G N K Q A V P
	BDG163	77-90	R V V P N G A G N K Q A V P
Epitope 4 (surface exposed)	Ref	370-395	NLTLCASTQNPVPSTYDPTKFKQYSR
	BDG08	370-395	NLTLCASTQTPVPNTYDPTKFKHYSR
	BDG22	370-395	NLTLCASTQNPVPNTYDPTKFKHYSR
	BDG163	370-395	NLTLCASTQNPVPGTYDPTKFKHYSR

Note: Amino acids highlighted with green were variable amino acids between epitopes. All predicted linear B-cell epitopes were shown in Suppl. 2.

## Discussion

*HPV 45 variant lineages*

Our recent study has shown that there were three nucleotide variations obtained of which one of these variations (sample BDG-163) is considered as a new L1 variant, since there is no HPV-45 L1 sequence that 100% identical with this isolate. The closest isolate with HPV-45 L1 sequence is Qv34178 from Costa Rica (GenBank accession number: EF202159.1) with 99.9% similarity. According to the HPV classification, a difference less than 2% within ORF of L1 with its closest isolate can be classified as HPV variant (Bernard et al., 2006; Bernard et al., 2005), however, other study has recommended to classify HPV variants based on its complete genome. With the definition of HPV variant, it should share >90% nucleotide identities, while intratype variant lineage should differ by 1-10% and intratype sublineage variant should differ by 0.5-1.0% (Burk et al., 2013).

Despite the discrepancy in classifying variant of HPV type, this minute finding in HPV-45 L1 nucleotide variation from Indonesia may indicate another variation in different region of the genome, since variation of nucleotide within a region in HPV genome may describe nucleotide variation in another region within its genome. HPVs are known to evolve through nucleotide changes, thus, nucleotide change in one region is highly correlate and attached to another changes in different regions within genomes from the same lineages (Chen et al., 2005). Moreover, from the phylogenetic tree, it appears that isolate BDG-163 has formed its own branch (Figure 1). Further study with complete genome analysis is needed to discover and to confirm whether this specific HPV-45 variant is from Indonesia.

Based on previous studies on HPV 16 and 18, it is suggested that the variant of HPV-16 and HPV-18 are correlated with geographic region from which they are isolated (Burk et al., 2009; Chen et al., 2005). However, the correlation between specific variant lineage and geographical region for other HPV genotype, including HPV-45, remains unanswered (Burk et al., 2013). It is well known that the variation within HPV genotype will have difference in viral persistence and cervical cancer development, for example, non-European HPV-18 variants has higher risk to develop cervical cancer, (de Oliviera et al., 2016; Bernard et al., 2005; Chen et al., 2014; Guan J et al., 2015) and similarly non-European HPV-16 variants also correlate with higher risk in progression to cervical cancer (Schiffman et al., 2010; Alfaro et al., 2016). The variants of HPV genotype other than HPV-16 and HPV-18 may also contribute to various risks in cancer development,(de oliviera et al., 2016; Chen et al., 2014) including HPV-45 as this genotype is the most closely related with HPV-18 (Chen et al., 2009). Hence, the geographical distribution of HPV-45 variants is diverse (Chen et al., 2014). Currently, the taxonomy of HPV-45 has been determined according to study by Chen et al., which classify HPV 45 into two main lineages A and B, and five sublineages A1, A2, A3, B1 and B2 (Chen et al., 2013). From this study, we found that four out of five HPV-45 from Indonesian isolates were grouped in a sublineage B2, which are known to be associated with higher risk of cervical cancer (Chen et al., 2014). Although in this study we used data of L1 HPV-45 sequence only from cervical cancer isolates (Table 1), and cannot compare the stage of cervical neoplasia, our finding could support previous study which found that lineage B2 is associated with higher risk of cervical cancer Chen et al., 2014). Interestingly, from histopathological data, four out of five samples are diagnosed as squamous cell carcinoma of cervix (Table 1), whereas HPV 45 are known to predominant in cervical adenocarcinoma (Quint et al., 2009; Sakamoto et al., 2018). Nevertheless, further research with more HPV-45 samples are needed to confirm this finding.

*Homology modelling of HPV-45 L1 and epitopes prediction*

The L1 protein is a major structural protein which forms a HPV capsid. On the surface of the capsid, there are 5 loops designated as BC, DE, EF, FG, and HI loop (Bisset et al., 2016). These surface exposed loops are known to be important for neutralizing antibody recognition, (Bisset et al., Buck et al., 2013) and sequence variation within the loops may affect efficiency of antibody neutralization (Godi et al., 2016; Guan J et al., 2015; Bisset et al., 2016). In this study, we have observed that the amino acid variation of HPV-45 L1 from Indonesian isolates are not showing major different in protein structure (Figure 2). Several mutations were found within surface region of L1, in correspond with the predicted linear B-cell epitopes (Table 3) along withn BC- and HI-loop (Figure 3). However, most of the amino acid mutations might not affect antibody neutralization since the polarity of altered amino acids are similar to mutation I329T. Although the polarity of amino acid has been changed, it is located within inner part of L1 monomer which is far from antibody recognition (Figure 4). Except for mutation S383G, which occur in surface loop of BDG-163 (HI-loop) this may be considered as new HPV-45 variant (Figure 3). The capsid of HPV is composed of L1 and L2, where DE- and FG- loops as the interface between L1 and L2 (Lowe et al., 2008). Therefore, it can be predicted that the conserved structure of DE- and FG- loops in HPV 45 L1 would not affect the overall structure of capsid. Further study using molecular dynamics simulation should be useful to explore the stability of binding between L1 and Fab, which reflecting the effect of mutations in L1 protein.

In conclusion, the HPV-45 from Indonesian isolates in this study are grouped into sub-lineage A2 and B2, with most of the isolates are clustered in B2 sub-lineage, which known to correlate with higher risk in developing cancer. Mutation in HI loop of new HPV-45 L1 variant results in an altered amino acid polarity found within the area of surface loop, suggesting a potential impact in antibody recognition and neutralization. Further study with more HPV-45 samples and exploration using molecular dynamics simulation are needed to reinforce these findings.
